# Progress of Surgery

**Published:** 1900-10-06

**Authors:** 


### Progress of Surgery.

### SURGERY OF THE STOMACH.

Frazier[1] gives an excellent summary of the literature of the Surgery of the Stomach, and Mayo Robson[2] in his Hunterian Lectures brings the whole subject up to date. According to Robson,[3] incision in the middle line between the xiphoid and umbilicus, although it gives the readiest access to the stomach in the greater number of cases, especially if there is a doubt as to what may be required, is not always the best site, for three reasons:—(1) It is apt to be followed by yielding of the scar and subsequent hernia unless long rest is enjoined subsequent to operation; (2) The round ligament of the liver with its irregular adipose envelope is apt to be in the way and prove confusing; (3) To prolong the incision for further investigation is inconvenient because of the umbilicus. These difficulties, of which the first only is of importance, are obviated by an incision through the inner margin of the rectus just to the right or left of the middle line. An incision on either side gives ample room, but if the pylorus is to be attacked it is preferable on the right; if gastro-enterostomy has to be done it is better on the left. The oblique incision along the costal margins is undesirable because it divides muscle so freely, and exact repair by suture is difficult. For these reasons Robson prefers the vertical incision through the inner edge of the rectus.

**Gastroptosis.**—Stengel and Beyea[4] report a case of Glenard's disease treated by operative procedure. The patient was a young unmarried woman, without history of traumatism, abdominal distension of any kind, or emaciation. The gastro-hepatic omentum and gastro-phrenic ligaments were exposed, separated from underlying structures, and interrupted sutures were introduced to shorten the gastro-hepatic omentum. Eleven months after the operation the woman was better in every way. The authors appear strongly inclined to recommend this operation in cases of gastroptosis in which there is abdominal pain, vomiting, and all the ill effects due to the enlarged stomach.

**Benign Obstruction of the Pylorus.**—Petersen[5] considers that pyloric stenosis with great motor deficiency, accompanied by emaciation and a permanent diminution of the quantity of urine, is an absolute indication for operative interference. When internal treatment fails, (1) severe atonic motor deficiency, (2) profuse hæmorrhages, and (3) severe gastralgia with uncontrollable vomiting, whether dependent on recent ulceration, cicatrices of ulcers, perigastritis, or adhesions, also demand surgical treatment. Great improvement in the mortality of such operations has taken place lately, and this he ascribes to greater experience in the choice of cases, and especially the substitution wherever possible of gastro-enterostomy or pyloroplasty for excision and resection, improved technique, especially the use of Murphy's button, which has reduced the time required for the operation from three-quarters to a quarter of an hour, allowed the patients to be fed by the mouth directly after the operation, lessened the chance of subsequent pneumonia, and abolished the danger of the formation of a "spur." Kammerer[6] divides the causes of benign obstruction of the pylorus into two classes, congenital and acquired. Under the first he considers the acute form, absolute at birth, and congenital hypertrophy of the pylorus. Among the most important of the acquired forms were fibrous stenosis, benign tumors, syphilitic gummata, gall stones obstructing the pylorus, and spastic contractions of the pylorus associated with hyperchlorhydria. In regard to treatment he emphasised the necessity of dietetics and of faithful lavage. These failing, surgical measures should be resorted to. Nicoll[8] reports a case of congenital stenosis of the pylorus which was relieved by Loreta's operation.

**Dilatation of the Stomach.**—The principal causes of this condition are thus classified by Bidwell[7]:—(1) Cicatricial contraction of the pylorus, due usually to old gastric ulcer; (2) Malignant disease of the pylorus; (3) Adhesions outside the pylorus; (4) Gastroptosis; (5) Spasm of the pylorus from ulcer or from excess of free hydrochloric acid; (6) Atony; (7) Various neuroses; (8) Floating kidney on the right side. The symptoms of malignant disease which would indicate surgical interference are:—(1) Pain, usually localised to the pyloric region and felt in the back; (2) Signs of dilatation of the stomach; (3) Retching and vomiting, usually only after solids; (4) Peristaltic waves seen over the stomach area; (5) Absence of free hydrochloric acid from the fluid obtained on passage of the stomach tube or from the vomited matter; (6) Progressive emaciation; (7) Obstinate constipation; (8) Progressive anæmia. The surgical procedures which are undertaken for the relief of dilated stomach vary with the cause. In malignant disease they are either pylorectomy or gastro-enterostomy; in non-malignant stenosis they are either pyloroplasty, gastro-enterostomy, Loreta's operation, pylorectomy, or division of adhesions. In gastroptosis and neurotic cases lavage and massage will usually suffice, but in obstinate or long-standing cases, where the dilatation does not yield to these means, the stomach may be fixed to the parietes (gastropexy) in gastroptosis, and in simple dilatation the capacity of the stomach can be diminished by folding in and suturing a portion of the anterior walls of the organ (gastroplication). Bennett[8] contributes an interesting clinical lecture on this subject.

**Gastrectomy.**—A successful case of complete resection of the stomach for carcinoma is reported by Harvie.[9] An instance of successful removal for epithelioma of the pylorus, together with adjacent portions of omentum which contained two glands in a state of advanced cancerous degeneration, is reported by Poirier.[10] The case is brought forward to show the fallacy of the opinion held by some pathologists that cancer of the stomach is a localised disease, and has but a slight tendency to invade the lymphatic system. Special attention should be paid to the small omentum; and the primary seat of disease, together with the suspected portions of omentum, should be removed in one mass, as is done in excision of mammary cancer, so that none of the degenerated lymph vessels are left.

**Gastric Ulcer.**—Huntingdon[11] reports some cases of this condition treated by surgical means, and quotes Lund's statistics as regards the most favourable time for operation. In a series of 45 cases operated upon within 12 hours after perforation 35 recovered—a mortality of 22 per cent. Of 70 cases operated upon within 24 hours 44 recovered—a mortality of 37 per cent. In a later series of 40 cases collected by the same author, 14 were operated upon within 12 hours, with 12 recoveries—a mortality of 14 per cent.; and 26, within 24 hours, with 19 recoveries—a mortality of 17 per cent.

[1] Amer. Jour. of the Med. Sci., May, 1900, p. 535. [2] Brit. Med. Jour., Mar. 10, Mar. 17, and Mar. 24, 1900; and Lancet, Mar. 31, 1900, &c. [3] Loc. cit. [4] Brit. Med. Jour., Aug. 7, 1900—Epitome No. 255. [5] Treatment, Jan. 25, 1900, p. 711. [6] Glasgow Med. Jour., April, 1900, p. 247. [7] Lancet, April 7, 1900, p. 999. [8] Brit. Med. Jour., Feb. 3, 1900, p. 241. [9] Annals of Surgery, Mar., 1900, p. 344. [10] Brit. Med. Jour., May 12, 1900—Epitome No. 331. [11] Annals of Surgery, April, 1900, p. 431.

### SURGERY OF THE PERITONEUM.

**Closing Abdominal Incisions** by through and through sutures is regarded by Richardson[1] as quicker, more convenient, less likely to leave dead spaces, and with no greater tendency to hernia than in other methods. He has adopted silkworm gut for closing the abdominal wall and silk for all other ligatures and sutures. It is questionable whether union is any more secure after suturing like tissues than if the peritoneum is sutured to the fascia and the fascia to the muscles. The number of herniæ through scars of abdominal incisions is insignificant. The objection to catgut is that it does not tie well and is not permanent enough. Silk never gives trouble in aseptic wounds.

**Diffuse Septic Peritonitis** is best treated, according to Fowler,[2] by drainage of the pelvis and elevation of the head of the bed. It has long been noted by surgeons that septic inflammatory processes when confined to the most dependent portion of the peritoneal cavity remain quiescent and without urgent symptoms for quite a period of time, as compared with a like condition of affairs existing in that portion of the peritoneal cavity situated above the pelvis. That infective processes in this region do not, as a rule, give rise to the same grave symptoms as an equal area of peritoneum infected in the abdominal cavity suggests the theory that the peritoneum in this region is possessed of qualities which enable it, first, to resist infection; second, to limit the infection within its own area when it does occur; and, third, either so to modify the virulence of the infection or to resist the absorption of its toxic products as to prevent the grave constitutional symptoms characteristic of an equally extensive infection of the peritoneum above the pelvis. In attaining these objects it is desirable that the elevation of the head of the bed from the horizontal shall be at least from twelve to fifteen inches. In order to prevent the patient from sliding down in the bed a large pillow is folded beneath the flexed knees, and upon this the buttocks rest. The pillow is prevented from sliding by a stout piece of bandage passed through at the folded portion and secured to the sides of the bedstead. In an interesting paper on the lymphatics of the peritoneum Robinson[3] says peritonitis is lymphangitis. On the behaviour of the lymphatics of the peritoneum towards invading infections depends the life of the subject. Should the lymphatics absorb the infectious invaders as rapidly as they would water, the patient would rapidly succumb to sepsis. Hence the peritoneal exudate which obstructs the lymphatics saves the subject from rapid death. Local peritoneal lymphangitis is chiefly due to muscular trauma. The pelvic lymphangitis is partially only due to the trauma of the psoas muscles. In the right iliac fossa the trauma of the psoas is responsible for appendicitis in the large number of cases. About the gall bladder the trauma of the diaphragm, right crux of the diaphragm, and the abdominal muscles tell the story. Careful microscopical examinations of pieces of the areas of local lymphangitis revealed the anatomic fact that the lymph vessels were almost entirely obliterated. Obliterating the lymph channels in the peritoneum left no means to transport the infectious material. In lymphangitis of the peritoneum the safety of the subject lies in obstruction of their lumen or external drainage.

**Vomiting** in its surgical aspects is considered by Bennett,[4] who concludes that: 1. Fæculent or stercoraceous vomiting occurs more commonly than is supposed in cases in which no mortal disease exists. The occurrence of fæculent vomiting is not always followed by death, even though nothing be done for the patient from a surgical point of view. 2. That in certain cases the vomiting is curative, inasmuch as it empties the bowel and stomach of accumulated contents which for the time are unable to find their way downwards in the normal way. 3. If, with the continuance of the vomiting there is persistent increase of abdominal distension, the condition is a mortal one unless radically relieved; but if with the continuance of the vomiting there is no increase of distension, there is reason to hope that surgical interference is unnecessary. 4. In cases of abdominal disease or injury fæculent vomiting as such is no positive indication for surgical interference, unless it is accompanied by increasing abdominal distension.

**Gun-shot Wounds of the Abdomen.**—Numerous reports from South Africa tell of severe abdominal injuries which have been recovered from without operation, and our estimate of the circumstances of abdominal surgery has been greatly altered by this war.[5] It does not seem at present possible to say what are the indications for operation; it is easier to point out the circumstances which preclude it. It may be said that inaction is demanded if the wound is a transverse or oblique one above the umbilicus, for it is then impossible to do all that is required. The openings in the bowel are many, the mesentery is likely to be pierced, and the liver may be wounded. If the bullet has entered the abdomen, and is there retained, there is no guide to its course, and operation would be purposeless. Wounds of the liver, kidney and colon alone are considered by Mr. Treves[6] to be unsuitable for interference, and he further states that most cases of wound below the umbilicus do well if left alone.

**Tuberculous Peritonitis.**—Elstem[7] has collected 227 cases of incision into the peritoneal cavity designedly for the cure of tuberculous disease. He concludes that the simple fact of incision is the most evident factor in cure in surgical proceedings of this class. Permanent cure after speedy disappearance of the local disease follows opening of the peritoneum without antiseptics, without washing out the peritoneal cavity, and without the aimless, useless, tedious, and therefore dangerous, dissecting away of tuberculous nodules, Fallopian tubes, &c.

[1] Brit. Med. Jour., June 2, 1900—Epitome No. 423. [2] Med. Record, April 14, 1900, p. 619. [3] Annals of Surgery, Feb., 1900, p. 214. [4] Brit. Med. Jour., Mar. 24, 1900, p. 693. [5] Brit. Med. Jour., June 9, 1900, p. 1424. [6] Ibid. [7] Ibid., April 28, 1900—Epitome No. 322.

